# Pregnancy-related acute kidney injury at high altitude: a retrospective observational study in a single center

**DOI:** 10.1186/s12882-021-02418-7

**Published:** 2021-06-09

**Authors:** Xin Li, Xiaojing Wu, Muyin Zhang, Lili Xu, Guohui Li, Yumei Wen, Weiming Wang

**Affiliations:** 1 Department of Nephrology, Shanghai Ruijin Hospital, Shanghai Jiaotong University School of Medicine, Shanghai, China; 2 Department of Internal Medicine, Diqing Tibetan Autonomous Prefecture People’s Hospital, Shangrila, China; 3 Department of Obstetrics and Gynecology, Diqing Tibetan Autonomous Prefecture People’s Hospital, Shangrila, China

**Keywords:** Acute kidney injury, Altitude, Hypertensive disorders of pregnancy, Pregnancy

## Abstract

**Background:**

Pregnancy-related acute kidney injury (Pr-AKI) is associated with maternal and fetal morbidity and mortality. There are few studies focusing on Pr-AKI at high altitude in the literature.

**Objectives:**

to investigate the incidence, etiology, clinical features and maternal-fetal outcomes of Pr-AKI in women living at high altitude.

**Methods:**

6,512 pregnant women attending the Department of Obstetrics & Gynecology at local hospital from January 2015 to December 2018 were screened for Pr-AKI. Patients with serum creatinine above normal range(> 70umol/L) then underwent assessment to confirm the diagnosis of Pr-AKI. AKI was diagnosed and staged based on Kidney Disease Improving Global Outcomes(KDIGO) guideline. Individuals meeting the Pr-AKI criteria were recruited. Their clinical data were recorded and retrospectively analyzed.

**Results:**

Pr-AKI was identified in 136/6512(2.09 %) patients. Hypertensive disorders of pregnancy(HDP) was the leading cause of Pr-AKI(35.3 %). 4(2.9 %) women died and the majority(86.1 %) had recovered renal function before discharge. Fetal outcomes were confirmed in 109 deliveries with gestational age ≥ 20 weeks. Pre-term delivery occurred in 30(27.3 %) cases and perinatal deaths in 17(15.5 %). The rate of low birth weight infant(LBWI) and intrauterine growth restriction(IUGR) was 22.0 and 10.9 % respectively. 16(14.5 %) infants were admitted to NICU after birth. Patients with HDP had a higher cesarean rate(56.3 %). More IUGR(25.0 %) and LBWI(37.8 %) were observed in their infants with a higher risk of admission to NICU(22.0 %). High altitude might have an adverse impact on HDP-related Pr-AKI patients with earlier terminated pregnancy and more stillbirth/neonatal death. Logistic regression models indicated that uncontrolled blood pressure, high altitude and advanced AKI were associated with adverse fetal outcomes in HDP-related Pr-AKI patients.

**Conclusions:**

Pr-AKI was not rare in high-altitude regions and caused severe fetal morbidities and mortalities. Uncontrolled blood pressure, high altitude and advanced AKI were all risk factors for adverse fetal outcomes in Pr-AKI patients, especially for those with hypertensive disorders of pregnancy.

**Supplementary Information:**

The online version contains supplementary material available at 10.1186/s12882-021-02418-7.

## Background

Pregnancy-related acute kidney injury(Pr-AKI) is an important obstetric complication, featured by a rapid decline in renal function and a series of consequential clinical disorders, making it one of the leading causes of maternal and fetal morbidity and mortality. As a heterogeneous disease with various underlying etiologies, it encompasses a wide range of clinical manifestations of different severity and can occur at any time of the pregnancy. Not only is Pr-AKI strongly associated with adverse outcomes of both mothers and their offspring, but also has a profound impact on their future health by increasing the risks of developing hypertension, chronic kidney disease(CKD) and cardiovascular diseases later in their lives[1, 2].

Over the last few decades, there has been a dramatic decrease in the overall incidence of Pr-AKI worldwide, especially in developing countries, largely owning to the promotion of antenatal supervision and the improvement in maternal-infant care. However, in areas where adequate antenatal care and sanitary facilities are not available, pregnancy-related diseases still remain a huge public health problem. Etiology spectrum has also changed. Hypertensive diseases, especially preeclampsia and its related complications, have replaced traditional causes of Pr-AKI, such as septic abortion and hemorrhage, becoming the predominant etiologies of AKI in pregnant women[3–5]. For women living at high altitude, there is another challenge. As the arterial oxygen saturation begins to decrease with ascending altitude, the risk of arterial hypoxia and its associated complications increases and may have a negative effect on pregnancy. Previous studies have found that long exposure to hypoxia increases the incidence of preeclampsia and other obstetric complications, including intrauterine growth restriction(IUGR), stillbirth and neonatal mortality[6–8], but further investigations and detailed demonstrations are needed to support these observations.

The incidence of Pr-AKI reported in Chinese population ranged from 0.02 to 1.84 % in recent years[3, 9], depending on different criteria they used to define AKI. However, most of the data came from tertiary hospitals in big cities. Data from undeveloped mountainous highlands was rare. Apart from social-economic factors, whether environmental factors play a role in the development of Pr-AKI and what influence they have on these patients deserve further discussion. Therefore, we conducted this research to investigate the incidence, etiology and clinical features of Pr-AKI at high altitude, and also looked into the risk factors of adverse pregnancy outcomes in these patients.

## Methods

### Subjects

A total of 6512 pregnant women were admitted to the Department of Obstetrics & Gynecology, Diqing Tibetan Autonomous Prefecture People’s Hospital, Shangrila, Yunnan province from January 2015 to December 2018. The hospital is located on the eastern edge of Qinghai-Tibet plateau, a vast mountainous region with an average altitude of 3,300 m(ranging from 1,504 m to 5,545 m) and 16 ethnic minorities. It serves as the only comprehensive medical institution in the region of Shangrila and receives patients from across this district as well as adjoining provinces including Tibet and Sichuan.

**Fig. 1:**
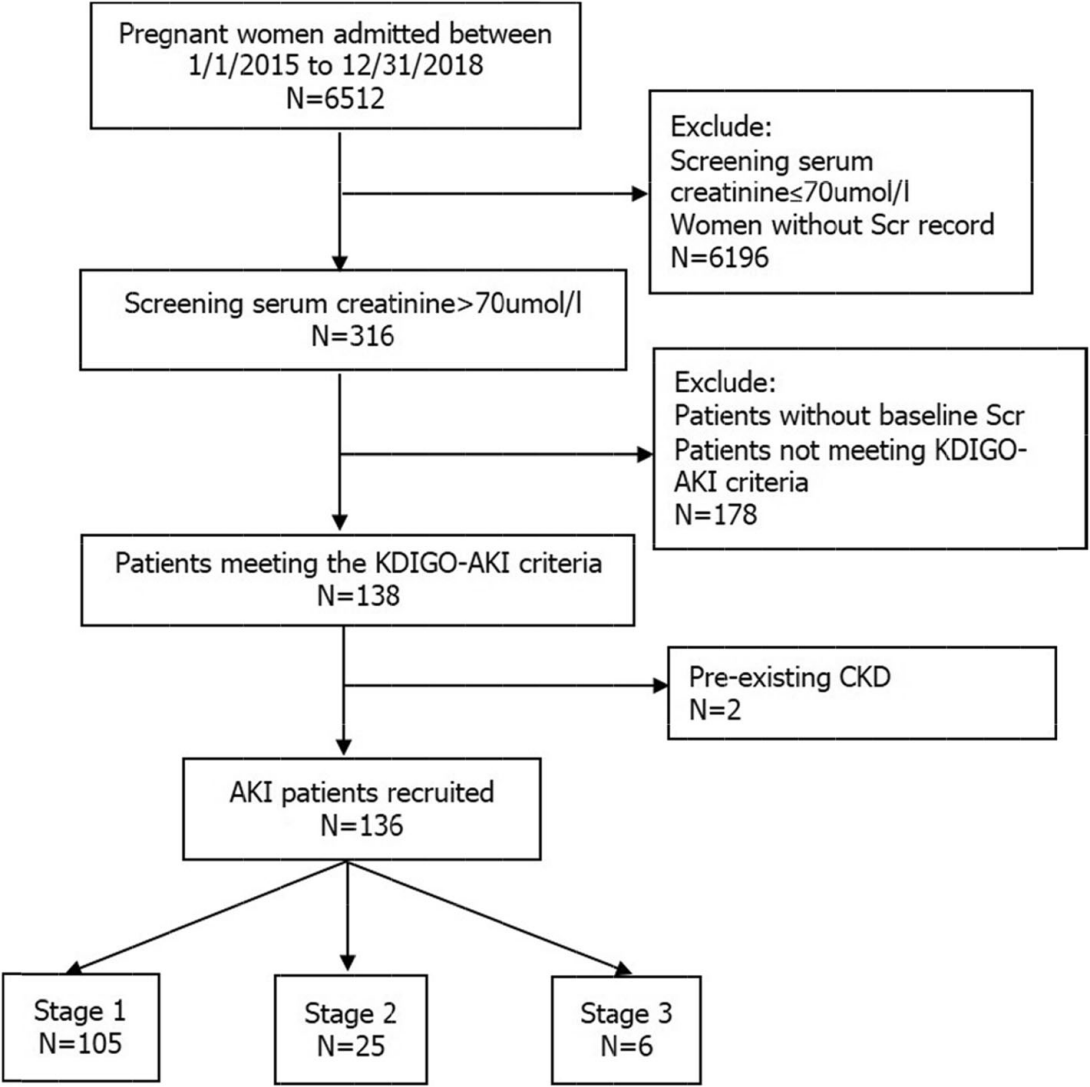
Flow chat of patient recruitment

We used an electronic medical record system to screen for candidates meeting the following characteristics: (1) Pregnancy or puerperium; (2) Diagnoses relevant to renal involvement including renal failure/insufficiency, proteinuria, chronic kidney disease(CKD) and renal imaging abnormalities such as kidney stone and hydronephrosis; (3) high-risk pregnancy-related conditions, which according to the previous literature, are associated with an increased risk of AKI during pregnancy and puerperium, including hypertensive disorders of pregnancy, preeclampsia, eclampsia, HELLP syndrome, sepsis, hyperemesis gravidarum, ectopic pregnancy, antepartum/postpartum hemorrhage, amniotic fluid embolism, pyelonephritis, acute fatty liver of pregnancy, heart failure and multiple organ dysfunction syndrome; (4) According to the existing literature, serum creatinine(Scr) > 70umol/L usually indicates renal dysfunction in pregnant women[10]. Therefore, Scr level>70µmol/l at least once during hospitalization was chosen as a threshold for the screening. After screening, all the candidates’ electronic medical records were reviewed by a nephrologist and an obstetrician respectively to confirm a diagnosis of Pr-AKI. Candidates were required to provide antenatal supervision within 3 months or physical examination records within 12 months before admission in order to assess their baseline renal function. Those without records of baseline renal function or with preexisting CKD were excluded. Finally, 136 patients meeting the Pr-AKI criteria with detailed clinical data for analysis were recruited in this study. Patient recruitment was summarized in Fig. 1.

### Data collection

Patients’ demographic and clinical information were registered, including: (1) demographic data: age, ethnicity, residential altitude; (2) medical history: records of antenatal supervision, previous obstetric history, complications; (3) clinical information: blood pressure, body mass index, gestational age on admission, singleton/multiple births; (4) laboratory and imaging findings: blood cell count, urine test, liver enzyme, serum creatinine, albumin, uric acid, lipid, urinary ultrasound; (5) pregnancy outcomes: delivery mode, gestational age at delivery/abortion, obstetric complications, maternal death. For patients with gestational week > 20weeks, fetal outcomes were also recorded, including: preterm delivery, stillbirth/neonatal death, birth weight, Apgar score at 1 min after delivery, intrauterine growth restriction(IUGR), admission to neonatal intensive care unit(NICU), low birth weight infants(LBWI)). Baseline renal function for patients having regular antenatal supervision were obtained from the lowest serum creatinine value available from their supervision records. For patients without regular antenatal supervision, serum creatinine measurements at primary/secondary hospitals or from physical examination records within 12 months would be adopted as the baseline value. For those without any records of biochemical tests before hospitalization, the most recent serum creatinine prior to the occurrence of Pr-AKI would be adopted as the baseline value after assessment of our team. We analyzed the most likely causes of AKI according to assessment of all the available clinical and laboratory evidence. Consecutive Scr measurements during hospitalization were recorded until death, discharge or the patients were transferred to another hospital.

### Related definition

Currently, there are no standardized criteria for the diagnosis of pregnancy-related AKI. Scr level typically is lower in pregnant women due to increased renal blood flow and glomerular hyperfiltration. Therefore, Scr level within normal range in non-pregnant women could reflect significant kidney injury in pregnancy. Criteria used in existing literature ranged from an increase in Scr to AKI requiring dialysis, among which the KDIGO guideline for AKI was the most widely used. In this study, we adopted the KDIGO guideline for Pr-AKI diagnosis and staging. According to the KDIGO guideline, an absolute increase in Scr level by ≥ 26.5µmol/l(0.3 mg/dl) within 48 h or a 50 % increase from the baseline value within 7 days is considered as AKI[11]. AKI stage was evaluated according to the ratios of maximum Scr to baseline Scr with a ratio of > 1.5, 2, and 3 denoting stages 1, 2, and 3, respectively. A rise of Scr ≥ 26 µmol/l within 48 h was considered as AKI stage 1, and a maximum Scr > 354 µmol/l was considered as AKI stage 3. Renal recovery was defined as final measured Scr<70umol/L before discharge.

The diagnosis of hypertensive disorders of pregnancy(HDP), preeclampsia, eclampsia was determined according to the definitions for hypertensive diseases from American College of Obstetricians and Gynecologists[12]. Diagnosis of HELLP syndrome, hyperemesis gravidarum, ectopic pregnancy, antepartum/postpartum hemorrhage, amniotic fluid embolism, pyelonephritis, acute fatty liver of pregnancy, heart failure and multiple organ dysfunction syndrome was made according to existing diagnostic criteria[13]. Term delivery was defined as delivery ≥ 37 weeks’ gestation. Preterm birth was defined as delivery<37 weeks’ gestation. Stillbirth was defined as fetal death after 20 weeks’ gestation. Neonatal death was defined when a newborn died before discharge from hospital. Low birth weight infant(LBWI) was defined as infants weighting less than 2500 gram. Intrauterine growth restriction(IUGR) was defined if birth weight of the newborn was below 10th percentile for given gestational age.

### Statistical analysis

Normally distributed continuous data were summarized using means ± standard deviation, and groups were compared using independent-sample t tests. Non-normally distributed data were summarized using medians(interquartile range) and compared across groups using Wilcoxon 2-sample tests. Categorical data were compared using Pearson χ2 test or Fisher exact test. Binary logistic regression model was used to explore the risk factors of adverse pregnancy outcomes. The statistical analysis was performed using SPSS version 22.0 for windows(SPSS Inc, Chicago, IL, USA). P < 0.05 was considered to be statistically significant.

## Results

### Incidence and timing of Pr-AKI

Pr-AKI was identified in 136/6512(2.09 %) pregnant women, approximately 1:48 pregnancies. 105(77.2 %) cases of Pr-AKI were stage 1 [median Scr 79µmol/L(75–88)]; 25(18.4 %) cases were stage 2[median Scr 116µmol/L(103-125.5)], and 6(4.4 %) cases were stage 3[median Scr 319.5µmol/L(173.8-438.8)]. Their demographic and baseline clinical data were showed in Table 1.

**Table 1:** Demographic and baseline clinical information of 136 patients with Pr-AKI

Variables	Results(N = 136)
Age (years)	27.7 ± 5.6
BMI (kg/m^2^)	24.9 ± 3.0
Residential altitude (m)	2804.4 ± 628.3
Primipara/multipara	76/60
Singleton/twin pregnancy	133/3
SBP (mmHg)	123.9 ± 28.9
DBP (mmHg)	80.6 ± 20.1
Hemoglobin (g/L)	116.4 ± 28.7
Platelet count (10^9^/L)	182.5 ± 91.2
ALT(IU/L)	14(11–25)
AST(IU/L)	20(16–29)
Serum ALB(g/L)	29.4 ± 7.8
SCr (umol/l)	84(75–98)
Serum UA (umol/L)	418.5 ± 159.9
Total glyceride(mmol/L)	3.8 ± 2.3
Total cholesterol(mmol/L)	5.6 ± 2.1

*AKI* acute kidney injury, *BMI* body mass index, *SBP* systolic blood pressure; *DBP* diastolic blood pressure; *ALB* albumin; *SCr* serum creatinine; *UA* uric acid; *ALT* alanine transaminase, *AST* aspartate transaminase

Among 136 Pr-AKI patients, 24(17.6 %) cases occurred in the first trimester, 10(7.4 %) in the second trimester, 91(66.9 %) in the third trimester and 11(8.1 %) during puerperium.

### Etiologies of Pr-AKI

Hypertensive disorders of pregnancy(HDP) was the leading cause of Pr-AKI and was responsible for 48(35.3 %) cases, followed by sepsis(33 cases, 24.3 %) and hemorrhage(23 cases, 16.9 %).(Supplement Table 1).

In the first trimester, post-abortion sepsis(*n* = 17) was the most common cause of Pr-AKI, followed by ectopic pregnancy(*n* = 7). Hemorrhage due to placenta previa and abruption of placenta was responsible for 5 cases in the second trimester. Pr-AKI caused by ectopic pregnancy(*n* = 2) and HDP(*n* = 2) was also noticed in this period. In the third trimester, HDP and its related diseases(*n* = 46), especially preeclampsia(*n* = 33), became the most common causes of Pr-AKI, while antepartum/postpartum hemorrhage(*n* = 16) and obstetric infections(*n* = 8) took the second and third place respectively. During the puerperium, most Pr-AKI cases were caused by puerperal sepsis(*n* = 9), and two cases were attributed to late postpartum hemorrhage due to retained placental tissues or dehiscence of cesarean section.

HELLP syndrome occurred in 3 patients with preeclampsia. 4 patients had acute fatty liver of pregnancy and 1 patient had amniotic fluid embolism. 3 cases were related to obstruction of urinary tract.

Besides the major cause, multiple factors could simultaneously contribute to the development of Pr-AKI as well. 49(36.0 %) patients had secondary or miscellaneous causes, including concomitant infections, insufficient renal perfusion and medication.

### Maternal and fetal outcomes of Pr-AKI

There were 4(2.9 %)cases of maternal death(2 patients with HELLP syndrome and 2 cases with rupture of uterus). No patients required emergency hemodialysis. Renal outcomes were confirmed in 115 patients (2 patients were transferred to other hospitals and 19 patients had no repeated Scr testing before discharge), and renal recovery occurred in 99(86.1 %) patients. Patients with stage 1 AKI had the highest renal recovery rate compared with patients with stage 2 and 3 AKI(93.1 % vs. 68.2 % vs. 50 %, *P* = 0.001).

Fetal outcomes were confirmed in 109 deliveries with gestational age ≥ 20 weeks, including two sets of twins. 42(38.9 %) patients delivered via cesarean section and the mean gestational age at delivery was 37.1 ± 3.3 weeks. The average birth weight and Apgar score-1 min after birth was 2935.2 ± 589.3 gram and 8.0 ± 3.3. Pre-term delivery occurred in 30(27.3 %) cases and perinatal deaths occurred in 17(15.5 %), including 8 stillbirths and 9 neonatal deaths. The rate of LBWI and IUGR was 22.0 %(24 cases) and 10.9 %(12 cases) respectively. 16(14.5 %) infants were admitted to NICU after birth due to prematurity-related complications.

### Pr-AKI in patients with hypertensive disorders of pregnancy

48 cases(including 2 twin pregnancies) of Pr-AKI were related to HDP(2 in the 2nd and 46 in the 3rd trimester), including 35 cases of preeclampsia, 2 cases of eclampsia and 3 cases of HELLP syndrome. Compared with 50 non-HDP patients(including 1 twin pregnancy), HDP-related Pr-AKI patients had a lower level of serum albumin(24.9 ± 5.6 vs. 27.5 ± 5.3, *P* = 0.02) and a higher level of uric acid(477.1 ± 82.8 vs. 406.2 ± 115.7, *P* = 0.01). Cesarean rate was much higher in HDP-related Pr-AKI patients than their counterparts(56.3 % vs. 31.3 %, *P* = 0.014). As for pregnancy outcomes, infants delivered by patients with HDP were more likely to have IUGR(25.0 % vs. 2.3 %, *P* = 0.003) or low birth weight(37.8 % vs. 15.9 %, *P* = 0.02), and also at higher risk of admission to NICU(22.0 % vs. 6.5 %, *P* = 0.019).(Table 2).

**Table 2:** Comparison of clinical features and pregnancy outcomes between HDP-related and non-HDP Pr-AKI patients

Variables	HDP-related *N* = 48 (50 fetuses)	Non-HDP *N* = 50 (50 fetuses)	*P* value
Age (years)	28.3 ± 5.1	26.9 ± 6.0	0.255
SBP (mmHg)	154.3 ± 19.0	110.6 ± 13.5	< 0.001
DBP (mmHg)	101.4 ± 15.3	69.3 ± 10.1	< 0.001
Serum ALB (g/L)	24.9 ± 5.6	27.5 ± 5.3	0.020
SCr (umol/L)	86(77–98)	90(78–111)	0.355^a^
Serum UA (umol/L)	477.1 ± 82.8	406.2 ± 115.7	0.001
Maternal death (n%)	2(4.0 %)	2(3.7 %)	1.000^b^
Gestational age at delivery (in weeks)	37.1 ± 2.8	37.0 ± 4.0	0.858
Delivery via cesarean section (n%)	27(56.3 %)	15(31.3 %)	0.014
Pre-term birth (n%)	17(35.4 %)	12(26.1 %)	0.328
Stillbirth/neonatal death (n%)	6(13.6 %)	10(21.3 %)	0.339
Neonatal weight (g)	2770.2 ± 647.4	3023.8 ± 523.7	0.054
Apgar score at 1 min	7.8 ± 3.3	7.7 ± 3.7	0.923
IUGR (n%)	11(25.0 %)	1(2.3 %)	0.003^b^
Admission to NICU (n%)	11(22.0 %)	3(6.5 %)	0.019^b^
LBWI (n%)	17(37.8 %)	7(15.9 %)	0.020
Renal recovery on discharge (n%)	29(76.3 %)	35(85.4 %)	0.305

*AKI* acute kidney injury; *HDP* hypertensive disorders of pregnancy; *SBP* systolic blood pressure; *DBP* diastolic blood pressure; *ALB* albumin; *SCr* serum creatinine; *UA* uric acid; *IUGR* intrauterine growth restriction; *LBWI* low birth weight infant; *NICU* neonatal intensive care unit

Values for categorical variables were given as number (percentage); values for continuous variables were given as mean ± standard deviation or median (interquartile range)

*P* < 0.05 was considered to be statistically significant

^a^: Wilcoxon 2-sample tests

^b^: Fisher exact test

### Impact of altitude on Pr-AKI patients

In order to assess the impact of altitude on patients with Pr-AKI, they were further divided into two groups according to their residential altitude: the low-altitude group(residential altitude<3000 m) and the high-altitude group(residential altitude ≥ 3000 m).

There were no statistically significant differences between the two groups in either clinical features or pregnancy outcomes(Supplement Table 2). However, in the HDP-related Pr-AKI subgroup, those living at high altitude had earlier terminated pregnancy(36.3 ± 3.1weeks vs. 38.0 ± 1.8weeks, *P* = 0.03) and more stillbirth/neonatal deaths(24.0 % vs. 0 %, *P* = 0.029) than their counterparts. Apgar-1 min scores(7.0 ± 3.8 vs. 8.8 ± 2.1, *P* = 0.091) and birth weight(2625.0 ± 694.4 g vs. 2963.9 ± 537.6 g, *P* = 0.093) were also lower compared with patients from low altitude in this subgroup.(Table 3).

**Table 3:** Subgroup analysis of clinical features and pregnancy outcomes between Pr-AKI women living at low and high altitudes

Variables	Without HDP (*N* = 50)			With HDP (*N* = 48)		
	LA *N* = 28	HA *N* = 22	*P* value	LA *N* = 21	HA *N* = 27	*P* value
Residential altitude (m)	2207.5 ± 345.4	3415.2 ± 244.4	-	2344.8 ± 199.7	3336.3 ± 146.9	-
Age (years)	27.6 ± 6.7	26.0 ± 4.6	0.333	27.9 ± 5.4	28.5 ± 5.0	0.686
BMI (kg/m^2^)	24.6 ± 2.4	24.2 ± 2.6	0.683	27.3 ± 5.2	27.3 ± 3.8	0.983
SBP (mmHg)	111.0 ± 14.9	110.6 ± 11.4	0.915	155.5 ± 18.2	153.8 ± 19.3	0.751
DBP (mmHg)	68.7 ± 10.2	69.6 ± 10.3	0.736	103.3 ± 13.6	101.5 ± 17.7	0.687
Hemoglobin (g/L)	105.4 ± 24.1	108.7 ± 38.6	0.718	125.5 ± 22.8	115.0 ± 29.9	0.187
Serum ALB (g/L)	27.4 ± 5.7	27.8 ± 4.7	0.826	25.7 ± 5.4	23.6 ± 6.0	0.212
SCr (umol/L)	91(76–109)	90(80–113)	0.872^a^	88(76–97)	86(77–100)	0.592^a^
Serum UA (umol/L)	429.1 ± 276.5	428.2 ± 112.5	0.989	481.4 ± 67.9	475.7 ± 91.5	0.809
Maternal death (n%)	1(3.4 %)	1(4.5 %)	1.000^b^	1(4.7 %)	1(3.7 %)	0.511^b^
Gestational age at delivery (in weeks)	36.9 ± 3.4	37.0 ± 4.6	0.890	38.0 ± 1.8	36.3 ± 3.1	0.030
Delivery by cesarean section (n%)	8(30.8 %)	7(31.8 %)	0.938	10(47.6 %)	17(63.0 %)	0.288
Pre-term birth (n%)	7(26.9 %)	5(25.0 %)	0.883	5(23.8 %)	12(44.4 %)	0.138
Stillbirth/neonatal death (n%)	6(23.1 %)	4(19.0 %)	1.000^b^	0	6(24.0 %)	0.029^b^
Neonatal weight (g)	3018.2 ± 580.1	3030.6 ± 461.8	0.942	2963.9 ± 537.6	2625.0 ± 694.4	0.093
Apgar score at 1 min	7.6 ± 3.8	7.8 ± 3.7	0.904	8.8 ± 2.1	7.0 ± 3.8	0.091
IUGR (n%)	0	1(5.3 %)	0.442^b^	3(20.0 %)	8(27.6 %)	0.480^b^
Admission to NICU (n%)	2(7.7 %)	1(5.0 %)	1.000^b^	5(27.8 %)	6(24.0 %)	1.000^b^
LBWI (n%)	5(20.8 %)	2(10.0 %)	0.428^b^	5(25.0 %)	12(48.0 %)	0.114
Renal recovery on discharge (n%)	22(91.7 %)	13(76.5 %)	0.212^b^	9(64.3 %)	20(83.3 %)	0.245^b^

*AKI* acute kidney injury; *HDP* hypertensive disorders of pregnancy; *LA* low altitude (<2500 m); *HA* high altitude (≥ 2500 m); *BMI* body mass index; *SBP* systolic blood pressure; *DBP* diastolic blood pressure; *ALB* albumin; *SCr* serum creatinine; *UA* uric acid; *IUGR* intrauterine growth restriction; *LBWI* low birth weight infant; *NICU* neonatal intensive care unit

Values for categorical variables were given as number (percentage); values for continuous variables were given as mean ± standard deviation or median (interquartile range)

*P* < 0.05 was considered to be statistically significant

^a^: Wilcoxon 2-sample tests

^b^: Fisher exact test

### Risk factors for adverse pregnancy outcomes

We used binary logistic regression models to explore potential risk factors for adverse pregnancy outcomes of Pr-AKI patients. Variables retained in the model were maternal age, residential altitude, AKI staging, and the highest systolic blood pressure after admission.

Among patients without HDP, only AKI staging was found associated with higher risk of stillbirth/neonatal death[OR 4.0(95 %CI 1.34,11.99), *P* = 0.013].

Among patients with HDP, high blood pressure was the strongest risk factor related to adverse fetal outcomes. For every 10mmHg increase in systolic blood pressure, the risk of preterm birth, LBWI, admission to NICU and IUGR would increase by 2.55 times[95 %CI(1.24,5.21), *P* = 0.011], 2.16 times[95 %CI(1.18,3.95), *P* = 0.013], 2.02 times[95 %CI (1.11,3.66), *P* = 0.21] and 1.75 times[95 %CI (1.03,2.99), *P* = 0.39] respectively. High altitude was found associated with elevated risk of LBWI[OR 7.30, 95 %CI(1.12,47.38), *P* = 0.037]. AKI staging was associated with higher risk of preterm birth[OR 17.6, 95 %CI(1.95,158.5), *P* = 0.011] and stillbirth/neonatal death[OR 11.5, 95 %CI(1.15,114.9), *P* = 0.038].(Table 4).

**Table 4:** Binary logistic regression models for potential risk factors of adverse pregnancy outcomes in Pr-AKI patients with and without HDP

Without HDP					
Variable	OR (95 %CI)				
	Preterm birth	Stillbirth/neonatal death	LBWI	NICU	IUGR
Maternal age (1 year)	1.10(0.97,1.26)	1.12(0.97,1.29)	1.02(0.89,1.18)	0.95(0.76,1.19)	NA
SBPmax (10mmhg)	0.69(0.41,1.16)	0.71(0.40,1.26)	1.29(0.66,2.53)	1.25(0.42,3.24)	NA
High altitude	1.06(0.24,4.74)	0.72(0.13,4.00)	0.39(0.06,2.59)	0.60(0.05,7.39)	NA
AKI staging	2.67(0.93,7.67)	4.00(1.34,11.99)	2.55(0.74,8.73)	0.87(0.11,6.88)	NA
With HDP					
Variable	OR (95 %CI)				
	Preterm birth	Stillbirth/neonatal death	LBWI	NICU	IUGR
Maternal age (1 year)	0.93(0.78,1.12)	0.95(0.71,1.27)	0.91(0.76,1.08)	0.92(0.77,1.10)	0.86(0.70,1.06)
SBPmax (10mmhg)	2.55(1.24,5.21)	0.87(0.39,1.93)	2.16(1.18,3.95)	2.02(1.11,3.66)	1.75(1.03,2.99)
High altitude(> 3000 m)	9.08(0.98,84.4)	NA	7.30(1.12,47.38)	1.21(0.24,6.13)	2.88(0.49,16.64)
AKI staging	17.6(1.95,158.5)	11.5(1.15,114.9)	5.06(0.65,39.39)	0.80(0.15,4.19)	3.98(0.68,23.21)

*AKI* acute kidney injury; *HDP* hypertensive disorders of pregnancy; *SBPmax* the highest systolic blood pressure after admission; *LBWI* low birth weight infant; *IUGR* intrauterine growth restriction; *NICU* neonatal intensive care unit; *NA* not applicable

*P* < 0.05 was considered to be statistically significant

## Comments

The landscape of pregnancy-related AKI has been changing dramatically over the last few decades. There has been a sharp decline in the overall incidence of Pr-AKI, not just in the developed world but also in the low-middle-income countries, mostly due to improvement in antenatal care and a significant decrease of septic abortion. The incidence of Pr-AKI in developed countries has remarkably reduced from approximately 1/3000 to 1/15,000–1/20,000 pregnancies from the 1960 s to the end of last century[14]. In developing countries such as India and China, the overall condition is also greatly improving. India has revealed a considerable decline in the proportion of Pr-AKI among hospitalized patients from 15.2 % in the 1980 s to 1.5–4.6 % in the 2010 s[15, 16]. In the meantime, maternal mortality has reduced to 5.79 % from initial high value 20 % in the 1980 s, and the progression of Pr-AKI to ESRD decreased to 1.4 % from 6.15 %[15].

As a developing country and also the world’s most populous nation, China has made impressive progress in fighting against severe obstetric diseases and reducing maternal-neonatal mortalities. The maternal mortality ratios have declined fast from 108.7/100,000 to 21.8/100,000 livebirths from 1996 to 2015[17], and the infant mortality has also decreased from 46.7 ‰ to 8.1 ‰ from 1992 to 2015[18]. In recent years, reported incidence of Pr-AKI in China is ranging from 0.02 to 1.84 %[3, 9], along with declining maternal and neonatal mortalities. Despite these encouraging achievements, huge regional discrepancies still exist within the country. In less economically developed western provinces, pregnancy-related diseases including Pr-AKI remain a critical public health problem to local people due to limited medical resources. According to the 2010 population census of China[19, 20], 58.6 % of the population in western China live in rural areas (eastern China: 40 %), 34.2 % of them belong to ethnic minority groups (eastern China: 2.1 %) and 10.1 % of women are illiterate (eastern China: 4.7 %). Underdevelopment, ethnic diversity, and geographical conditions all present great challenges to ensuring universal access to medical resources. In our study, the incidence of Pr-AKI in the region of Shangrila was 2.09 %, higher than previous data reported by other Chinese authors. Given that we had excluded patients who delivered out of hospital as well as those unable to provide baseline renal function, this figure was likely to be by far underestimated.

There is also an urgent need to raise the awareness of Pr-AKI among medical practitioners in local medical institutions. Pr-AKI is not rare among women of childbearing age, and the incidence can be extremely high in patients with particular obstetric complications. It affects approximately 5-17 % of patients with severe preeclampsia[4, 9, 21] and 7-60 % of patients with HELLP syndrome[9, 22, 23]. However, a recent study revealed that only 4.0 % of AKI events were initially diagnosed on the discharge record[24]. Patients at early stage of renal impairment sometimes present with Scr levels within normal range due to physiological glomerular hyperfiltration. In our study, the median Scr level was 84umol/l(0.95 mg/dl). Only 24(17.6 %) patients had a Scr level > 105umol/l(1.1 mg/dl). In the setting of pregnancy, any Scr value above 70.72umol/l (0.8 mg/dl) should be considered abnormality. That’s why we used 70umol/l as a cut-off value for screening Pr-AKI candidates in this study. The relative change of Scr within a time period should also be taken into account, rather than just using a fixed upper limit value when making an AKI diagnosis. Judging by Scr levels alone will miss mild AKI events in patients with low baseline Scr, leading to underestimation in clinical practice.

In general, maternal outcome in less severe Pr-AKI is favorable. However, severe Pr-AKI is associated with significantly increased mortality, especially among those requiring dialysis. Recent studies from China and India report that maternal mortality rate associated with severe Pr-AKI ranges from 4.0 to 8 %[3, 17, 25]. In the long run, approximately 4-9 % of women with severe Pr-AKI requiring dialysis will remain dialysis-dependent for another 4–6 months after delivery[26]. The rate of progression to end-stage renal disease from Pr-AKI ranges from 1.4 to 2.5 %[17, 27].

Pr-AKI also presents a harsh challenge for the newborns. Perinatal mortality including intrauterine death, stillbirth and neonatal death occurs in 23.5-54 % of Pr-AKI patients[5, 25], and the odds of perinatal mortality increase 3.39 fold when compared with pregnancies without Pr-AKI[27]. Pr-AKI is also linked to preterm delivery and disturbance of fetal development, resulting in IUGR, low birth weight or infant small for gestational age. This trend is particularly significant in patients suffering from HDP or preeclampsia. Prematurity and small babies are not only associated with prenatal death and fetal distress, but also have potential long-term detrimental impacts. Preterm is a strong risk factor for development of CKD in adulthood because of the delayed and insufficient kidney growth which may result in a low nephron number[28]. Additionally, it has been demonstrated that CKD, small and preterm babies will be at high risk for development of diabetes, metabolic syndrome and cardiovascular diseases in adulthood[29, 30].

We also looked into the impact of high altitude on pregnant women, especially those with HDP. Previous literature has reported a correlation between long exposure to high altitude and adverse fetal outcomes. The odds of stillbirth and preterm increase by 3.9-fold and 1.7-fold respectively at high altitude relative to low altitude[31]. High altitude is also associated with lower birth weight, reducing fetal birth weight by an average of 102 g for every 1000 m increase in elevation[32]. These findings can be partially explained by maternal arterial hypoxia from high altitude exposure. In addition to this, long exposure to high altitude can interfere with effective transportation of oxygenated blood to the utero-placental circulation. High altitude decreases the pregnancy-induced increase in cardiac output and blood volume, likely due to a failure to reduce peripheral vascular resistance[33]. It also interferes with maternal vascular adjustment to pregnancy, such as the growth and remodeling of the uterine artery and other utero-placental vessels, attenuating the normal increase of uterine artery diameter and blood flow[34]. It also alters patterns of blood flow redistribution, for example, by reducing the proportion of common iliac blood flow distributed to the uterine versus the external iliac artery, resulting in lower uterine artery blood flow near term[34, 35]. In the presence of preeclampsia, which is commonly accompanied by utero-placental vascular dysfunction and placental ischemia, insufficient maternal arterial oxygenation and compromised compensatory mechanism from long exposure to high altitude will further aggravate placental insufficiency and fetal intrauterine hypoxia. Moreover, long exposure to high altitude is also responsible for the pathogenesis and development of preeclampsia. The incidence of preeclampsia increases by approximately 3-fold at high altitude[36]. Hypoxia regulates the production of numerous vascular endothelial regulatory factors known to influence vascular function during pregnancy and/or to be associated with preeclampsia, including endothelin-1, thromboxane A2, prostacyclin and tumor necrosis factor alpha[37]. Animal experiments also confirm that long-term high altitude hypoxia during gestation suppresses large conductance Ca2+-activated K+(BKCa) channel function in uterine arteries, which is of critical importance in pregnancy-mediated increase in uterine artery vasodilation and blood flow, resulting in the dysfunction of uterine placental circulation and the development of preeclampsia[38]. Hypoxia can also activate placental renin-angiotensin system, leading to placental dysfunction and oxidative stress response, which enhance the vasoconstrictor effect of locally-generated angiotensin II and contribute to the restricted fetal growth characteristic of preeclampsia[39, 40]. As preeclampsia has proved to be an important contributory factor to adverse fetal outcomes, we believe that the interaction between hypoxia and preeclampsia may have synergistic effects and enhance the adverse impacts of preeclampsia on pregnancy outcomes.

### Strengths and limitations

Clinical researches focusing on AKI within pregnancy, especially for women from high altitude are rare, and the association between environmental factors including altitude and pregnancy outcomes in Pr-AKI patients is scarcely discussed in the literature. In this study, we made a comprehensive analysis of the clinical data of Pr-AKI patients in the high-altitude regions of western China and also looked into the risk factors of adverse pregnancy outcomes in these people. We found that uncontrolled blood pressure, high altitude and advanced AKI were all risk factors for adverse fetal outcomes in HDP-related Pr-AKI patients, while in Pr-AKI patients without HDP, only AKI stage was found associated with worse fetal outcomes. Our finding emphasized the importance of blood pressure control among hypertensive pregnant women, as we revealed a strong connection between uncontrolled blood pressure and almost all types of adverse outcomes in their fetus. There might also be a link between high altitude and adverse fetal outcomes including stillbirth/neonatal death and LBWI especially in HDP-related Pr-AKI patients. It highlighted the necessity of intensive perinatal care and monitoring for patients from high altitude in the presence of HDP and advanced AKI stages, and called for more reasonable distribution of social and medical resources to patients at high risk in areas with limited resources. With more than 140 million people living at high altitude all around the world and the high prevalence of HDP among pregnant women in less economically developed regions, this finding was of great clinical and social-economic significance.

Our study has some limitations. Firstly, histological data were unavailable because renal biopsy couldn’t be performed due to technological limitations of the local hospital. Etiologies of Pr-AKI that relied on renal biopsy might be misdiagnosed or get overlapped with pre-existing underlying renal diseases which were commonly seen in women of childbearing age, for example, IgA nephropathy and lupus nephritis. The second limitation is the incomplete follow-up records. Pregnancy outcomes of critically ill patients who had to be transferred to tertiary hospitals located at lower altitudes were not included in our analysis. Besides, most patients lost to follow-up after discharge, making it difficult to assess their long-term renal outcomes. Thirdly, confounding factors that might be related to pregnancy outcomes such as nutritional status, income and education levels were not analyzed in this study due to the limitation of data sources and small sample size. Finally, since our data came from a single-center study with a small group of patients, studies with enlarged sample size or even multi-center prospective cohorts were required to validate our findings.

## Conclusions

In summary, this study revealed that Pr-AKI was not rare in high-altitude regions. It affected around 2.09 % of the pregnant women but this figure might be by far underestimated. Although the overall maternal outcome was favorable, it still caused severe fetal morbidities and mortalities. More attention should be paid to patients with HDP, because it was not only the main cause of Pr-AKI but also led to worse pregnancy outcomes compared with patients with other etiologies. Uncontrolled blood pressure, high altitude, and advanced AKI were all risk factors for adverse fetal outcomes in HDP-related Pr-AKI patients. Thus more intensive and timely perinatal care and monitoring were needed for high-risk patients in order to improve their prognosis.

## Supplementary Information

**Additional file 1:** Etiologies of Pr-AKI in different phases of pregnancy

**Additional file 2:** Comparison of clinical features and pregnancy outcomes between Pr-AKI patients from low and high altitude

## Data Availability

The datasets generated and analyzed during the current study are available from the corresponding author on reasonable request.

## Abbreviations

Pr-AKI: Pregnancy-related acute kidney injury
KDIGO: Kidney Disease Improving Global Outcomes
HDP: Hypertensive disorders of pregnancy
BMI: Body mass index
SBP: Systolic blood pressure
DBP: Diastolic blood pressure
ALB: Albumin
SCr: Serum creatinine
UA: Uric acid
ALT: Alanine transaminase
AST: Aspartate transaminase
IUGR: Intrauterine growth restriction
LBWI: Low birth weight infant
NICU: Neonatal intensive care unit
