# Supplementary material for: Pregnancy-related acute kidney injury at high altitude: a retrospective observational study in a single center
Source: BMC Nephrol. 2021 Jun 9;22:215. doi: 10.1186/s12882-021-02418-7 (PMC8190839; doi:10.1186/s12882-021-02418-7)
Supplement: Supplementary file 1 — Additional file 1: Etiologies of Pr-AKI in different phases of pregnancy [file 12882_2021_2418_MOESM1_ESM.docx]

Supplementary table 1. Etiologies of Pr-AKI in different phases of pregnancy

| Causes of Pr-AKI | Total  N=136 | 1^st^ trimester  N=24 | 2^nd^ trimester  N=10 | 3^rd^ trimester  N=91 | Puerperium  N=11 |
| --- | --- | --- | --- | --- | --- |
| HDP | 48(35.3%) | 0 | 2(1.5%) | 46(33.8%) | 0 |
| Sepsis | 33(24.3%) | 16(11.8%) | 0 | 8(5.9%) | 9(6.6%) |
| Antepartum hemorrhage | 9(6.6%) | 0 | 4(2.9%) | 5(3.7%) | 0 |
| Postpartum hemorrhage | 14(10.3%) | 0 | 1(0.7%) | 11(8.1%) | 2(1.5%) |
| Ectopic pregnancy | 9(6.6%) | 7(5.1%) | 2(1.5%) | 0 | 0 |
| Acute fatty liver of pregnancy | 4(2.9%) | 0 | 0 | 4(2.9%) | 0 |
| Dehydration | 3(2.2%) | 1(0.7%) | 0 | 2(1.5%) | 0 |
| Amniotic fluid embolism | 1(0.7%) | 0 | 0 | 1(0.7%) | 0 |
| Hydronephrosis | 3(2.2%) | 0 | 1(0.7%) | 2(1.5%) | 0 |
| Others | 12(8.8%) | 0 | 0 | 12(8.8%) | 0 |

HDP: hypertensive disorders of pregnancy
