# Supplementary material for: Pregnancy-related acute kidney injury at high altitude: a retrospective observational study in a single center
Source: BMC Nephrol. 2021 Jun 9;22:215. doi: 10.1186/s12882-021-02418-7 (PMC8190839; doi:10.1186/s12882-021-02418-7)
Supplement: Supplementary file 2 — Additional file 2: Comparison of clinical features and pregnancy outcomes between Pr-AKI patients from low and high altitude [file 12882_2021_2418_MOESM2_ESM.docx]

Supplementary table 2.Comparison of clinical features and pregnancy outcomes between Pr-AKI patients from low and high altitude

| Variables | Low altitude  N=49 (50 fetuses) | High altitude  N=49 (50 fetuses) | P value |
| --- | --- | --- | --- |
| Age (years) | 27.7±6.2 | 27.4±4.9 | 0.746 |
| BMI (kg/m2) | 25.8±4.1 | 26.0±3.6 | 0.791 |
| Residential altitude(m) | 2266.3±297.4 | 3371.7±198.5 | - |
| SBP (mmHg) | 130.2±27.6 | 134.8±27.0 | 0.401 |
| DBP (mmHg) | 83.6±20.9 | 87.5±21.8 | 0.366 |
| Hemoglobin (g/L) | 113.9±25.4 | 112.2±33.7 | 0.784 |
| Serum ALB (g/L) | 26.7±5.6 | 25.3±5.8 | 0.252 |
| SCr (umol/L) | 90(76-101) | 86(79-103) | 0.484^a^ |
| Serum UA (umol/L) | 451.7±213.2 | 455.4±102.7 | 0.913 |
| Maternal death (n%) | 2(4.1%) | 2(4.1%) | 1.000^b^ |
| Gestational age at delivery  (in weeks) | 37.4±2.9 | 36.7±3.8 | 0.280 |
| Delivery by cesarean section (n%) | 18(38.3%) | 24(49.0%) | 0.292 |
| Pre-term birth (n%) | 12(25.5%) | 17(36.2%) | 0.264 |
| Stillbirth/neonatal death (n%) | 6(13.3%) | 10(21.7%) | 0.292 |
| Neonatal weight (g) | 2993.8±554.9 | 2798.8±632.6 | 0.143 |
| Apgar score at 1 min | 8.1±3.2 | 7.4±3.7 | 0.326 |
| IUGR (n%) | 3(7.1%) | 9(20.0%) | 0.082 |
| Admission to NICU (n%) | 7(15.9%) | 7(15.6%) | 0.963 |
| LBWI (n%) | 10(22.7%) | 14(31.1%) | 0.373 |
| Renal recovery on discharge (n%) | 31(81.6%) | 33(80.5%) | 0.902 |

AKI: acute kidney injury; BMI: body mass index;SBP: systolic blood pressure; DBP: diastolic blood pressure; ALB: albumin; SCr: serum creatinine; UA: uric acid; IUGR: intrauterine growth restriction; LBWI: low birth weight infant; NICU: neonatal intensive care unit

Values for categorical variables were given as number (percentage); values for continuous variables were given as mean ± standard deviation or median (interquartile range)

P<0.05 was considered to be statistically significant

a: Wilcoxon 2-sample tests

b: Fisher exact test
